# Experimental study on the mechanical characteristics of weakly cemented mudstone under different loading rates

**DOI:** 10.1038/s41598-024-65024-1

**Published:** 2024-07-04

**Authors:** Junpeng Zou, Gang Li, Zibo Li, Yabing Zhang, Hang Liu, Yiming Wang

**Affiliations:** 1 Collage of Mining, Liaoning Technical University, Fuxin, 123000 China; 2 School of Safety Engineering, Heilongjiang University of Science and Technology, Harbin, 150022 China

**Keywords:** Weakly cemented mudstone, Rate effect, Uniaxial acoustic emission, Mechanical properties, Floor heave, Civil engineering, Petrology

## Abstract

With the gradual shift of coal mining to the western coal mining region of China, floor heave in weakly cemented mudstone roadways has become an issue affecting the safety and efficiency of coal mine production. Additionally, different mining rates can lead to fluctuating support stresses on the roof and floor of weakly cemented mudstone roadways. Therefore, obtaining a comprehensive understanding of the mechanical properties of weakly cemented mudstone at different loading rates is conducive to improving the issue of floor heave in such roadways and provides a theoretical basis for further study. In this context, a series of uniaxial mechanical tests with concurrent acoustic emission monitoring were conducted on specimens of weakly cemented mudstone under various loading rates (0.005, 0.01, 0.05, and 0.1 mm/s). The stress‒strain and acoustic emission response curves were obtained to effectively characterize the strength, deformation, damage, macroscale instability, and crack propagation characteristics of the mudstone under the influence of loading rate effects. The research results support the following findings: (1) With increasing loading rate, the peak strength and elastic modulus of weakly cemented mudstone significantly increase, while the peak axial strain and peak radial deformation significantly decrease. (2) With increasing loading rate, the stress required to trigger the expansion of weakly cemented mudstone gradually increases, and a significant power-law relationship arises between the strain of the mudstone at the start of expansion and the loading rate. (3) With increasing loading rate, the acoustic emission ringing count of weakly cemented mudstone increases: The failure of weakly cemented mudstone changes from small-range progressive failure to sudden failure, and the failure mode transitions from shear failure to tensile‒shear composite failure. (4) The studied mudstone damage variables increase with increasing loading rate, following an approximate exponential function. The conclusions obtained in this work can provide a theoretical basis for the evolution mechanism and control of floor heave in deep roadway mining.

With the gradual shift of coal resources to deeper depths, the problem of floor heave in roadways has become increasingly prominent. Floor heave has gradually become an important factor affecting the safe mining of coal^1^. Kang^2^ considered rock dilation to be an important factor that causes floor heave, which is closely related to the properties and stress state of the rock. Rock dilation was first observed by Bridgman^3^ in 1949, and many experts have conducted research on dilation since then. Whether rock undergoes dilation depends on the physical properties of the rock and the stress state it experiences; in particular, expansion may be dependent on deviatoric stress and plastic strain^4,5^. Liu^6^ classified the dilation stage based on the uniaxial compression failure stage of the rock, stating that dilation damage in the yielding stage of sandstone significantly increases. It is believed that rock dilation can serve as an indicator of the rock failure process. In previous studies^7,8^, the stress range for the dilation point of the rock was found to be 0.6–0.85 σ_max_.

Under the action of external loads, rock failure is attributed to damage and volume expansion caused by cracking^9^. The mechanical properties of rocks depend on the extension and coalescence of internal microcracks^10,11^. Additionally, the stress environment has a certain influence on crack propagation in rocks. Huang^12^ conducted laboratory tests on rock specimens with one and two cracks under two different unloading conditions and revealed that the crack propagation process is intermittent. Chen^10^ investigated the dilation characteristics of sandstone under different confining pressures. When a specimen is tested under higher confining pressure conditions, more cracks tend to develop before failure. Simultaneously, with increasing confining pressure, the dilation strain and crack propagation velocity of sandstone increase.

In the process of roadway excavation, different excavation rates^13–17^ and cyclic loading induced by roof fracture^18^ have significant effects on rock damage. Studies^19–22^ have investigated the energy evolution patterns of rocks under different loading rates, revealing a proportional relationship between plastic strain and loading rate and inverse relationships between residual stress, plastic capacity, and dissipation energy and loading rate. Research^23^ on the mechanical properties of coal under various loading rates suggests that the sensitivity of the mechanical behaviour of coal to nonuniform loading tends to decrease with increasing loading rate. Other research^24^ has suggested that as the loading rate increases, crack propagation shifts from a high-energy-consumption low-energy-efficient fracture mode to a low-energy-consumption high-energy-efficient fracture mode. A transition in the rock failure mode from tensile failure to shear failure with increasing loading rate has been observed^25^. In summary, the dilation characteristics of rocks are determined by their mineral composition, pore structure, and stress environment. The loading rate significantly influences the mechanical properties of rocks, and current research primarily explores the effects of the loading rate on rocks through compression tests. The loading rate and its variations essentially reflect the viscous characteristics of the rocks. The existing rock testing standards do not yet have a completely unified loading rate criterion. Typically, strain rates in the range of 10^–5^–10^–1^/s fall within the static range, while rates exceeding 10^–1^/s are considered dynamic^26,27^. For strain rates below 10^–5^/s, creep effects should be considered^28^.

Currently, research on the dynamic response of rocks is mostly conducted under high strain rates. Moreover, studies on the loading-rate-dependent behaviour of rocks are primarily focused on hard and brittle rocks, with limited research on soft rocks such as mudstone. This study targets the floor mudstone of the Yuecheng Coal Mine in Jincheng city, Shanxi Province. After thoroughly analysing the mineral composition and microstructure of this weakly cemented mudstone, uniaxial compression experiments were conducted at different loading rates. The mechanical properties of the weakly cemented mudstone under different loading rates were further analysed from the perspectives of acoustic emission and damage. This research aims to provide a theoretical basis for the stability control of deep roadway surrounding rock.

## Physical properties of the rock

### Source of the rock samples

**Figure 1:**
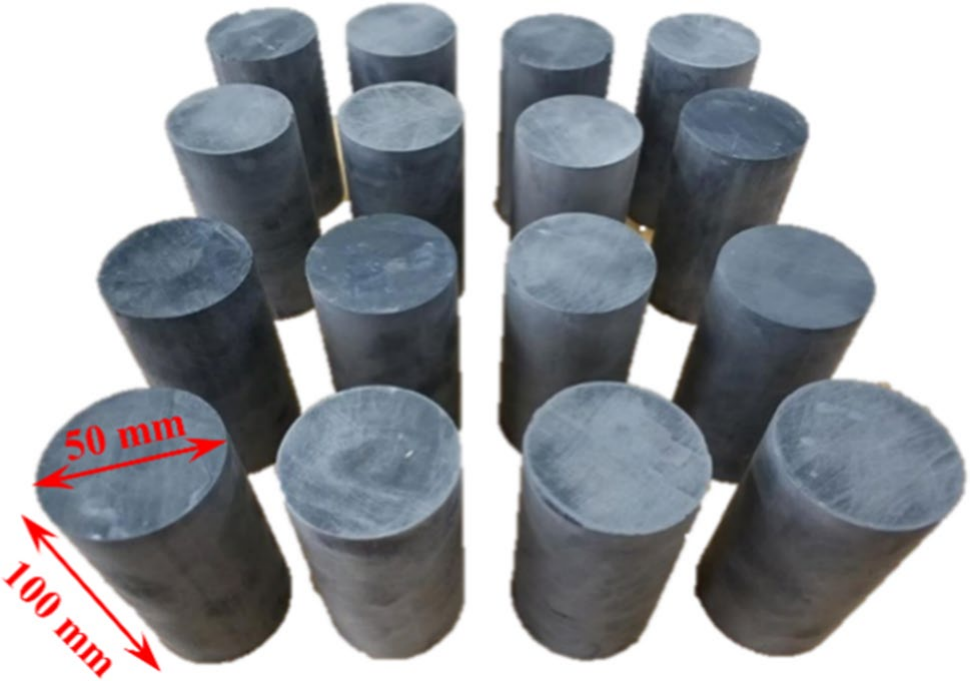
Rock samples.

The specimens for this study were sourced from the Yuecheng Coal Mine in Jincheng city, Shanxi Province. The mine has a production capacity of 120 Mt/a, and the coal-bearing strata belong to the Upper Taiyuan Formation of the Carboniferous System. The primary coal seam for extraction is the 15th coal seam. Samples were collected from the mudstone underlying the 15th coal seam, which predominantly consists of grey mudstone. The samples were obtained at a depth of 500 m. Due to difficulties in on-site drilling, samples larger than 300 mm were collected in the field and subsequently processed in the laboratory using a water-free cutting method to obtain standard specimens with a diameter of 50 mm and a height of 100 mm, following the methods outlined in the "Methods of Testing for Coal and Rock Physical Mechanics". The rock specimens were ground using a rock grinder to achieve standard specimens with parallelism less than 0.05 mm. To minimize the influence of rock fractures on the experimental results, a sonic velocity instrument was used to select rock samples with similar longitudinal wave velocities for testing. In this study, rock specimens with a wave velocity ranging from 1800 to 2000 m/s were chosen, as illustrated in Fig. 1.

### X-ray diffraction analysis of the rock mineral composition

**Figure 2:**
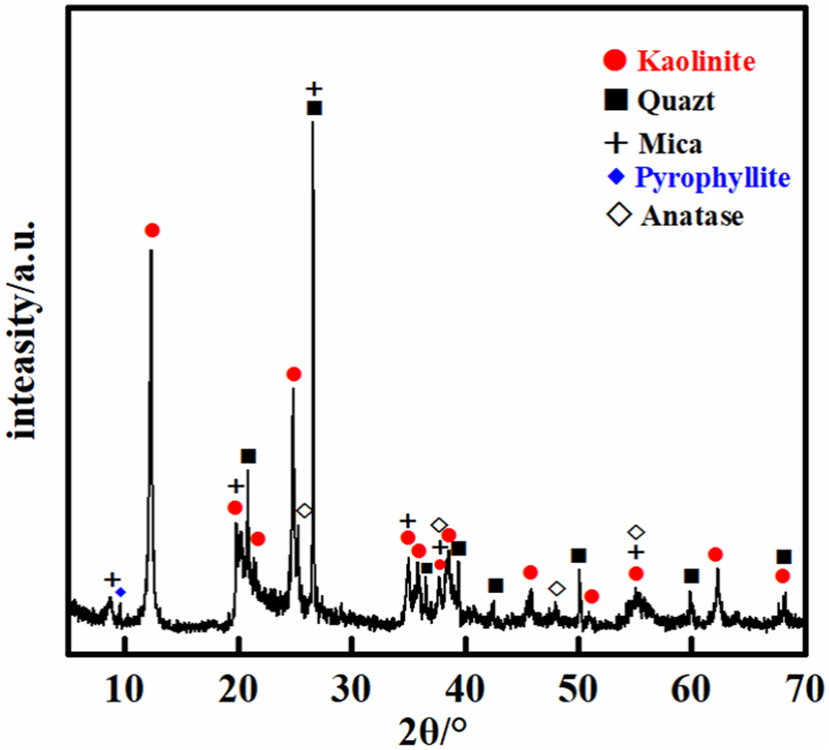
XRD mineral test results.

To analyse the mineral composition of the mudstone, X-ray diffraction (XRD) was employed for sample analysis. The experimental results are shown in Fig. 2. The mudstone is primarily composed of clay minerals, with kaolinite being the predominant mineral, accounting for approximately 80% of the mudstone. Additionally, the mudstone contains minerals such as quartz, anatase, kaolinite, and mica.

### Rock porosity structure characteristics

**Figure 3:**
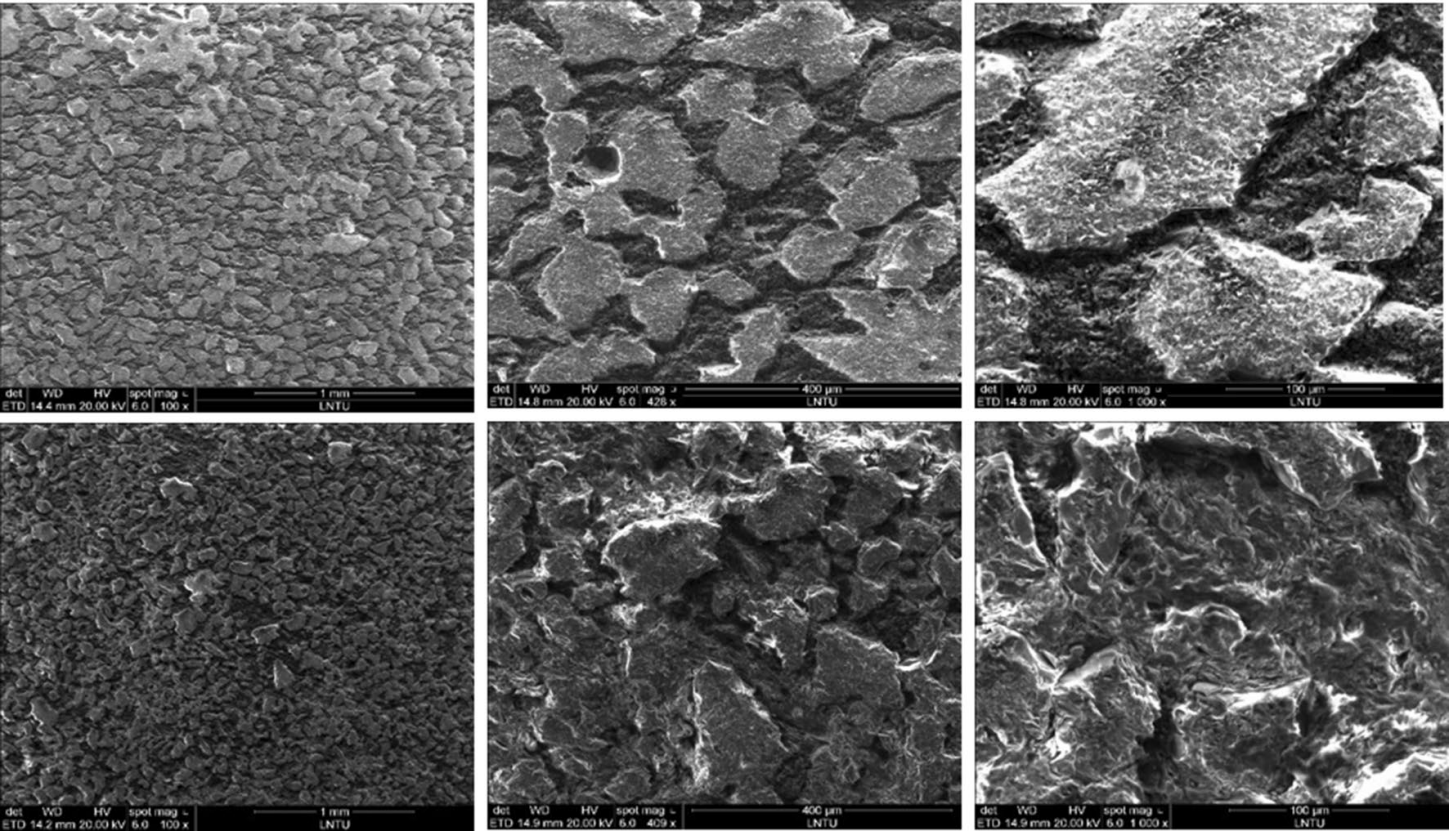
SEM images of the mudstone.

To observe the porosity and structural characteristics of the mudstone, scanning electron microscopy (SEM) was performed on an FEI Quanta TM 250 instrument, and SEM images were obtained at various magnifications, as shown in Fig. 3. From the images, it is evident that the distribution of the components of the mudstone is nonuniform. The primary component (kaolinite) in the mudstone exhibits a network-like distribution, indicating a cemented network structure. The mudstone contains significant pores and fractures.

## Experimental apparatus and procedures

**Figure 4:**
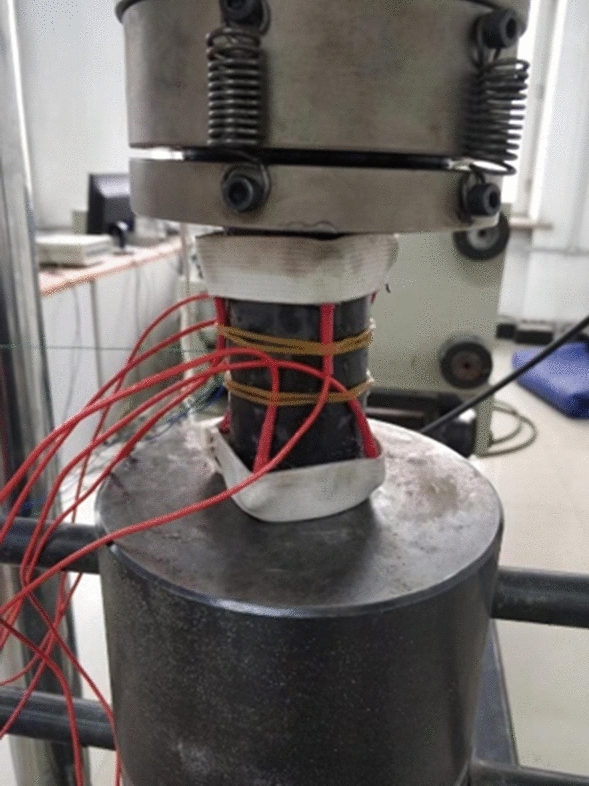
Installation position of the acoustic emission probe.

In this experiment, the TAW-2000 microcomputer-controlled electrohydraulic servo rock triaxial testing system and SH-II acoustic emission system were utilized for uniaxial compression tests under different loading rates. The equipment has an axial loading range of 0–2000 kN, a measurement accuracy of 0.001 kN and a resolution of 0.001 mm. The loading displacement was set to 40 mm, and the force loading rate ranged from 0.001 to 1 mm/s. The strain gauge used was the uT7150A static strain gauge produced by Wuhan Youtai Company, with a strain range of 0 to ± 30,000 με and a monitoring accuracy of 0.1 με. Acoustic emission monitoring was conducted using an SH-II acoustic emission instrument with a threshold of 35 dB. The arrangement of the acoustic emission probes is illustrated in Fig. 4; the probes were uniformly distributed radially along the rock at a distance of 15 mm from the top and bottom surfaces.

For this experiment, loading rates of 0.005, 0.01, 0.05, and 0.1 mm/s were applied to the samples to obtain their stress‒strain curves and acoustic emission characteristics under different loading rates. Prior to an experiment, a Nano30 sensor with a resonance frequency close to that of rock at failure was used to coat the surface with Vaseline. The acoustic emission probe was then securely fixed to the rock surface with a rubber band. An acoustic emission automatic testing system and a break lead experiment were employed to verify its monitoring effectiveness.

## Experimental results

### Stress‒strain curves

**Figure 5:**
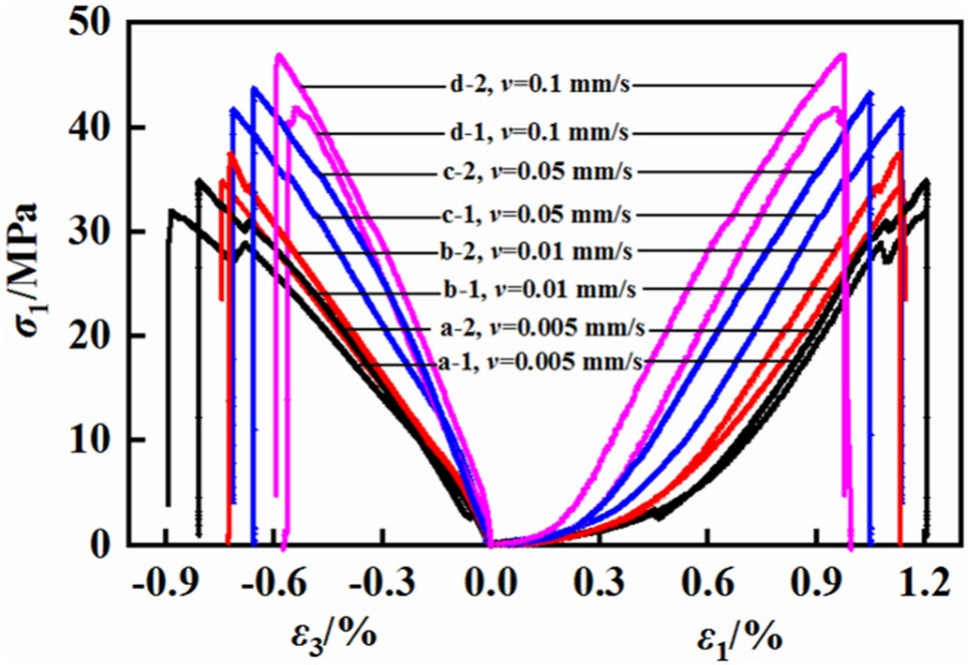
Stress‒strain curves under different loading rates.

This experimental work was conducted at four different loading rates, and the results are presented in Table 1. In Table 1, 'v' represents the loading rate, and the uniaxial compressive strength (UCS) of the mudstone varies from a minimum value of 29.83 MPa to a maximum of 46.91 MPa. The elastic modulus ranges from a minimum of 4.91 GPa to a maximum of 6.83 GPa. Figure 5 shows the stress‒strain curves of the mudstone specimens under the different loading rates tested. Figure 5 clearly shows that during the loading process, the rock exhibits elastic‒plastic characteristics, with each specimen undergoing a compacted phase with crack closure. As the loading rate increases, the compaction phase becomes shorter. Upon entering the elastic phase, the rock's slope and elastic modulus increase with increasing loading rate. In the plastic phase, the rock experiences varying degrees of damage, and a higher loading rate results in a less distinct plastic phase.

**Table 1:** UCS test results of mudstone specimens under.

v/mm s^−1^	Number	*σ*_UCS_ (MPa)	*ε*_1_	*ε*_3_	*E* (GPa)
0.005	a-1	31.87	1.207	− 0.892	5.45
	a-2	34.83	1.206	− 0.809	4.91
	Average value	33.35	1.2065	− 0.8505	5.18
0.01	b-1	34.8	1.15	− 0.746	5.53
	b-2	37.47	1.13	− 0.726	5.25
	Average value	36.14	1.14	− 0.736	5.39
0.05	c-1	41.74	1.13	− 0.755	6.47
	c-2	43.66	1.05	− 0.695	5.97
	Average value	42.70	1.09	− 0.725	6.22
0.1	d-1	41.94	0.994	− 0.575	6.83
	d-2	46.91	0.977	− 0.574	6.6
	Average value	44.43	0.9855	− 0.5745	6.715

### Strength characteristics

**Figure 6:**
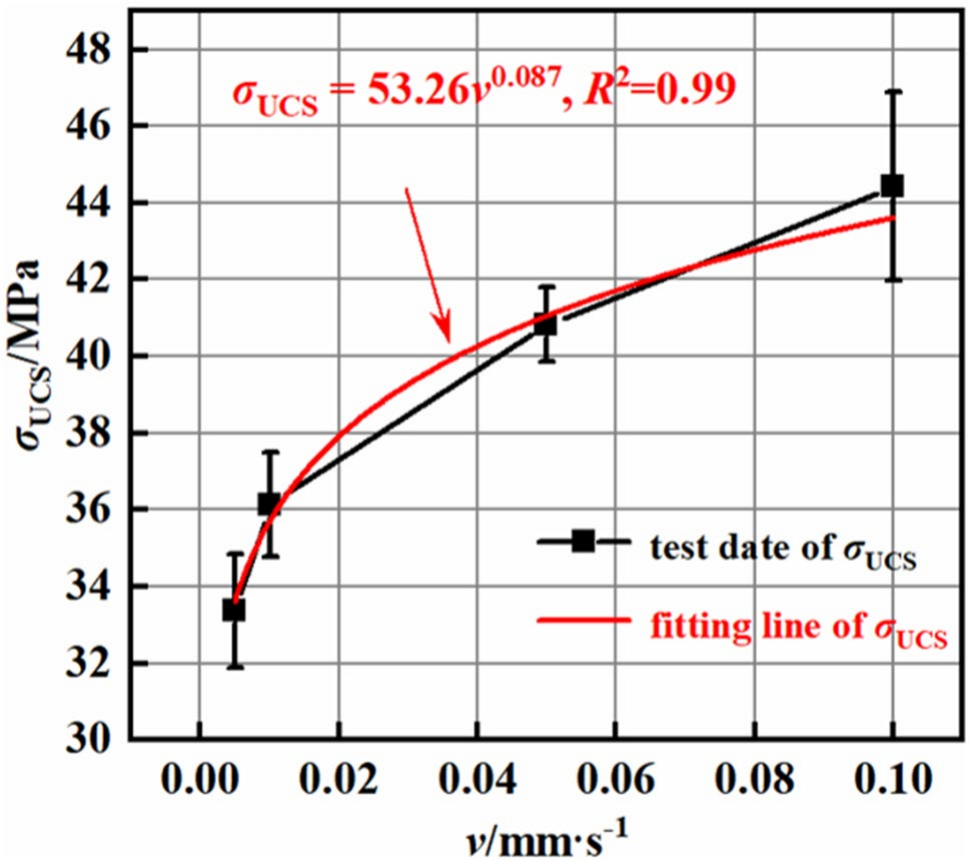
UCS–loading rate curves.

**Figure 7:**
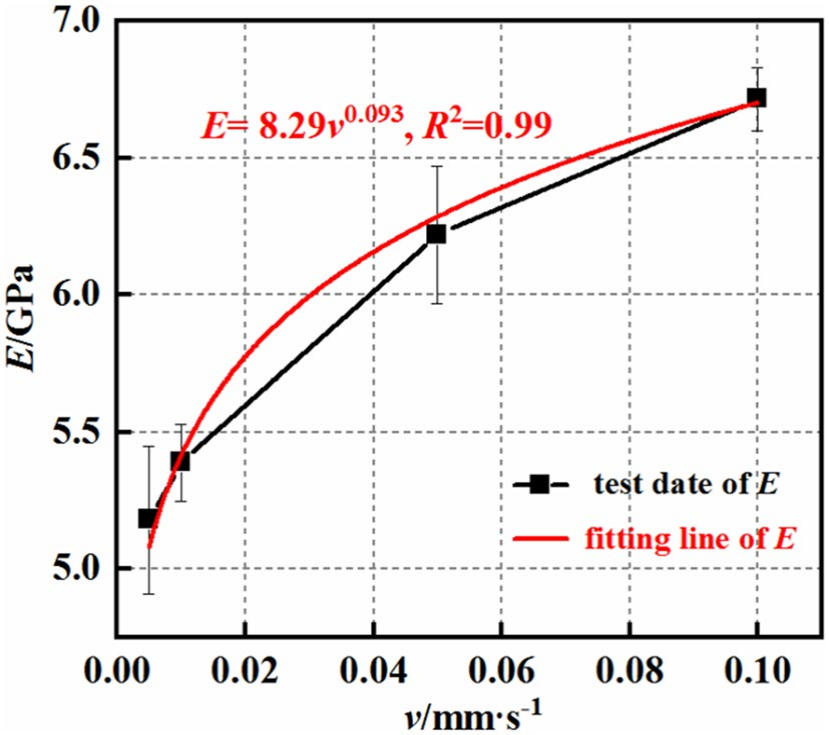
Elastic modulus–loading rate curves.

Figures 6 and 7 illustrate the variations in the peak strength (σ_ucs_) and elastic modulus (E) of the rock at different loading rates (V). Figure 6 depicts the relationship between the peak strength of the mudstone and the loading rate. The peak strength of the rock increases with increasing loading rate, and the increase in peak strength is proportional to the loading rate, following a power function ${\upsigma }_{{{\text{ucs}}}} = {53}{\text{.26}}\;{\text{v}}^{{{0}{\text{.087}}}}$. The fitted equation has a correlation coefficient of 0.99. Since the data used for the fitted equation in this paper only includes experimental results for loading rates from 0.005 to 0.1 mm/s, the fitted equation in this paper is only applicable to weakly cemented mudstone with loading rates from 0.005 to 0.1 mm/s^29,30^. When the loading rate increases by 2, 10, and 20 times, the average peak strength of the rock increases by 8.37, 28.04, and 33.19%, respectively. This clearly demonstrates that an increase in the loading rate leads to an increase in the peak strength of the rock. Moreover, the magnitude of the increase in peak strength is smaller than the multiplier of the loading rate, indicating that the increase in peak strength has a certain limit. Furthermore, as the loading rate increases, the rate of increase in the peak strength gradually decreases. Figure 7 presents the relationship between the elastic modulus of the rock and the loading rate. The elastic modulus of the rock gradually increases with increasing loading rate, following a power function ${\text{E}} = {8}{\text{.29}}\;{\text{v}}^{{{0}{\text{.093}}}}$. The fitted equation has a correlation coefficient of 0.99. When the loading rate increases from 0.005 s to 0.1 mm/s, the average elastic modulus of the rock increases from 5.18 to 6.715 GPa. With a 20-fold increase in the loading rate, the elastic modulus increases by 29.6%. As with its strength, the elastic modulus of mudstone increases with increasing loading rate. However, the magnitude of increase decreases with increasing loading rate, indicating that the loading rate can influence only the peak strength and elastic modulus of mudstone to a certain extent. Additionally, as the loading rate increases, the impact becomes less significant.

### Deformation characteristics

**Figure 8:**
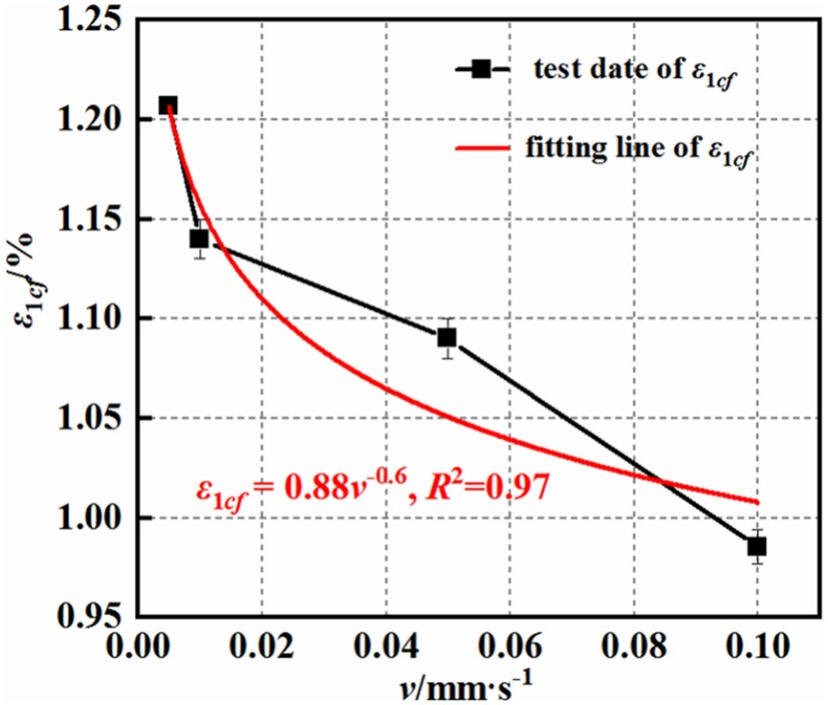
Maximum axial strain–loading rate curves.

**Figure 9:**
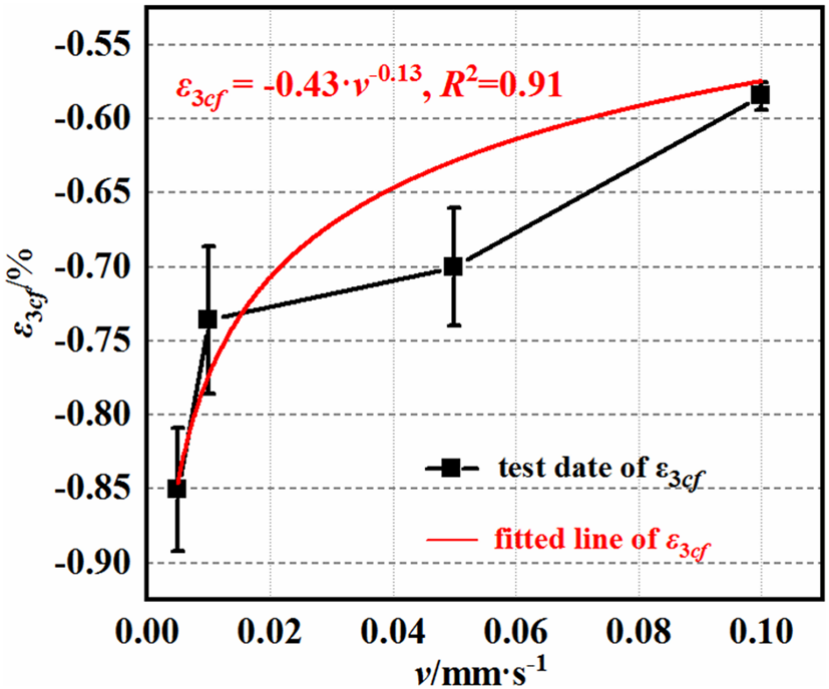
Maximum radial strain–loading rate curves.

Figures 8 and 9 illustrate the deformation characteristics of the mudstone under different loading rates. Here, ε_1cf_ represents the average maximum axial deformation of the mudstone, and ε_3cf_ represents the average maximum radial deformation. The axial and radial deformations of the mudstone are influenced by the loading rate. As the loading rate increases, both the axial and radial strains gradually decrease. The average maximum strain exhibits a power function relationship with the loading rate. The axial strain is represented by ${\upvarepsilon }_{1{{\text{cf}}}} = {0}{{.88}}\;{\text{v}}^{{ - {0}{{.6}}}}$, with a correlation coefficient of 0.97, and the radial strain is represented by ${\upvarepsilon }_{3{{\text{cf}}}} = - 0.43{\text{v}}^{{ - {0}{{.13}}}}$, with a correlation coefficient of 0.91. When the loading rate increases from 0.005 to 0.01 mm/s, 0.05, and 0.1 mm/s, i.e., a 2-fold, 10-fold, and 20-fold increase, respectively, the axial strain decreases by 5.5, 9.6, and 18.3%, respectively. In contrast, the radial strain increases by 13.4, 14.8, and 32.5, respectively. The influences of the loading rate v on the axial and radial strains differ in magnitude, indicating that the impacts of the loading rate on the axial and radial strains gradually diminish as the loading rate increases.

## Mudstone dilatancy characteristics

### Dilatancy deformation characteristics

The dilatancy characteristics of mudstone were calculated with the axial and radial strains from the stress‒strain curves obtained during uniaxial compression tests. The calculation formula is as follows:

1 $${\upvarepsilon }_{{\text{v}}} = {\upvarepsilon }_{{1}} + {{2\upvarepsilon }}_{{3}}$$

where ε_v_ is the volumetric strain, expressed as a percentage; ε_1_ is the axial strain (generally positive); and ε_3_ is the radial strain (generally negative).

**Figure 10:**
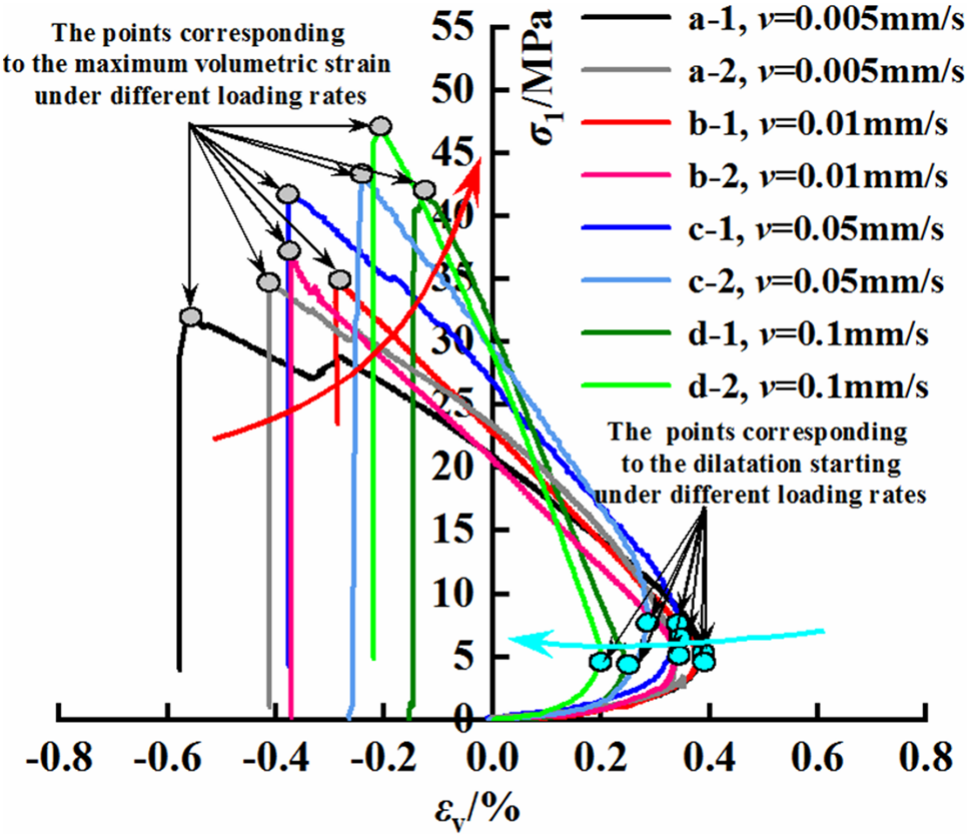
Volumetric strain–loading rate curves.

This experimental work was conducted under four different loading rates: 0.005, 0.01, 0.05, and 0.1 mm/s. The resulting stress‒volumetric strain curves under different loading rates are shown in Fig. 10. Under various loading rates, all the samples exhibit dilatancy characteristics. In the initial stages of loading, there is a noticeable slow increase in volumetric strain, and a larger loading rate corresponds to a slower initial increase in volumetric strain. This phenomenon may be attributed to the initial compaction of the rock fractures. Subsequently, the volumetric strain gradually increases, with a higher loading rate resulting in a faster increase in volumetric strain, indicating the end of the rock fracture compaction stage. After reaching the dilatancy point, the volumetric strain of the mudstone gradually decreases. The rate of decrease in the volumetric strain after the dilatancy point increases with the loading rate, with a steepening slope of the curve. Moreover, as the loading rate increases, the maximum volumetric strain of the mudstone gradually decreases, suggesting that at higher loading rates, the mudstone experiences failure before undergoing sufficient deformation. In this experiment, due to the high content of weathered minerals in the selected mudstone, the mudstone has a lower strength and higher porosity. Therefore, under the tested loading rates, the deformation of the mudstone exhibits strong expansion characteristics, which depend on the mineral composition and pore structure of the mudstone. Compared with those of sandstone, the expansion characteristics of this weakly cemented mudstone are more significant.

### Dilatancy strength characteristics

**Figure 11:**
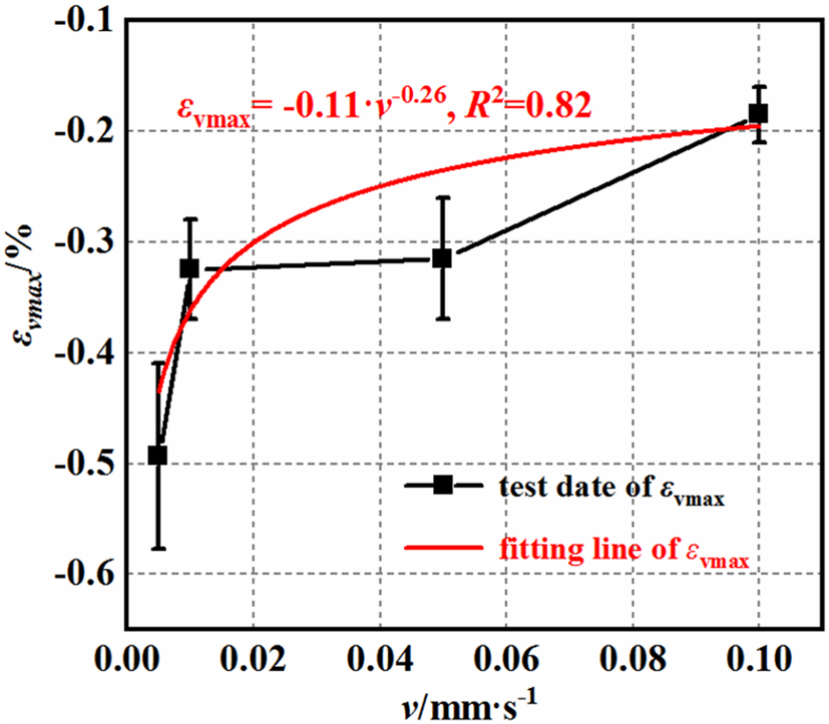
Maximum volumetric strain–loading rate curves.

Here, the dilation initiation stress (σ_c_), dilation initiation strain (ε_c_), and maximum volumetric strain (ε_vmax_) of the mudstone were separately calculated. As shown in Fig. 11, the relationship between the maximum volumetric strain of mudstone and the loading rate follows a power function ${\upvarepsilon }_{{{\text{vmax}}}} { = } - {0}{{.11}}\;{\text{v}}^{{ - {0}{{.26}}}}$, with a correlation coefficient of 0.82. This indicates that as the loading rate increases, the maximum volumetric strain of the mudstone gradually decreases. The rate of decrease diminishes over time, approaching a constant value. This suggests that the influence of the loading rate on the volumetric strain is limited and tends to stabilize with increasing loading rate.

**Figure 12:**
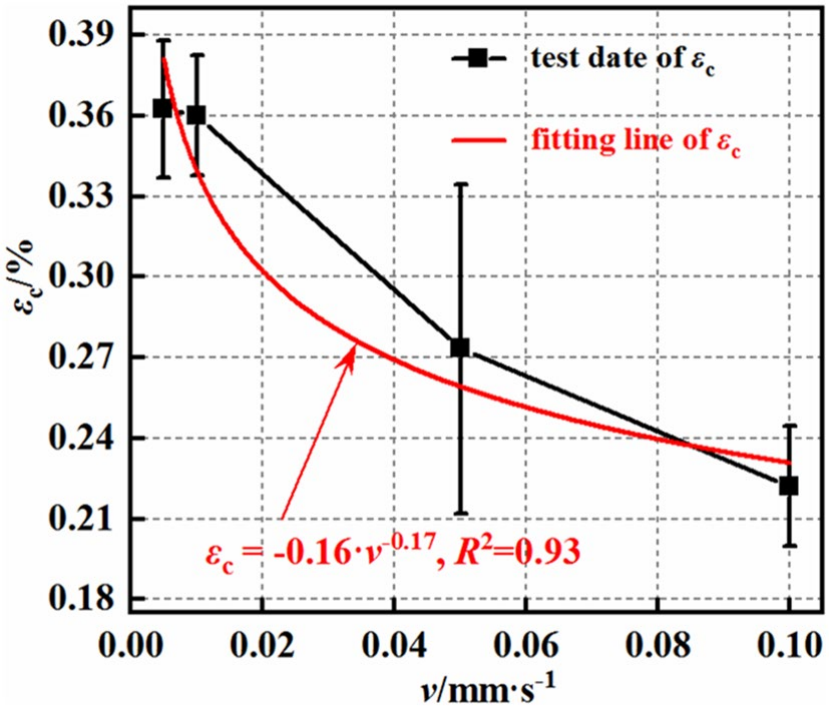
Dilation initiation strain–loading rate curves.

Figure 12 shows that the dilation initiation volumetric strain of the mudstone decreases with increasing loading rate. The dilation initiation strain of mudstone also exhibits a power function relationship with the loading rate, expressed as $\varepsilon_{{\text{v}}} = - 0.16\;{\text{v}}^{ - 0.17}$, with a correlation coefficient of 0.93. This indicates that as the loading rate increases, the dilation initiation strain of the mudstone gradually decreases. When the loading rate of the mudstone increases by 2, 10, and 20 times, the dilation initiation volumetric strain decreases by 5.5, 9.6, and 18.3%, respectively.

**Figure 13:**
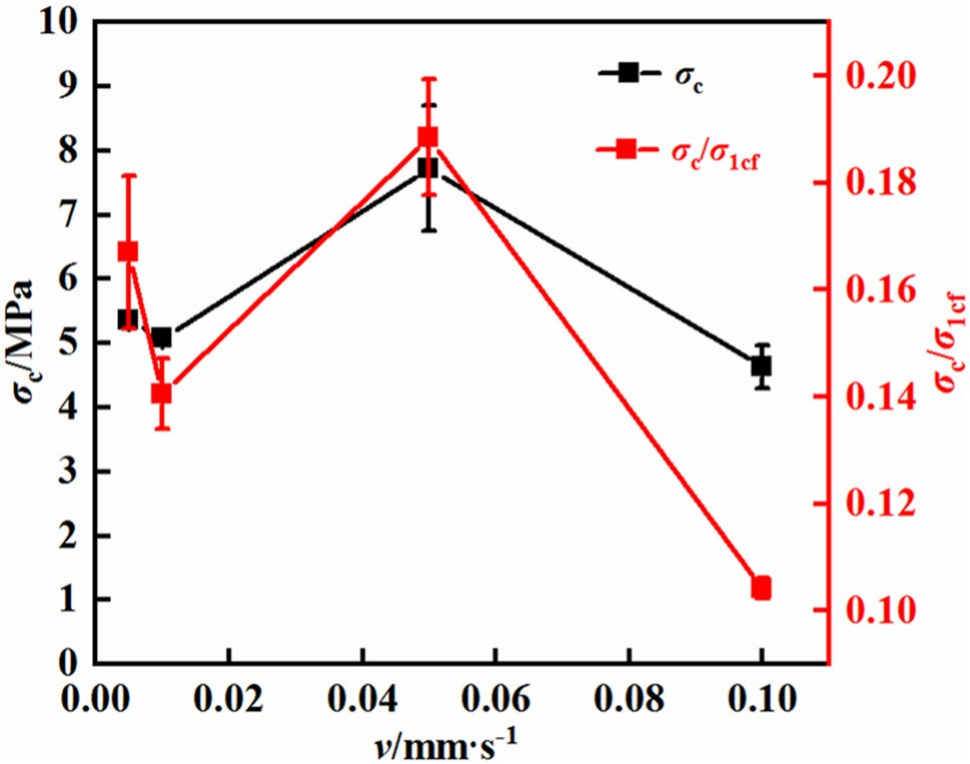
Dilation initiation stress–loading rate curves.

Figure 13 shows the relationship between the dilation initiation stress of the mudstone and the loading rate. With increasing loading rate, the dilation initiation stress of mudstone significantly changes. When the loading rate of the mudstone increases by 2, 10, and 20 times, the dilation initiation stress decreases by 5.5%, increases by 49.5%, and decreases by 13.7%, respectively. Moreover, the ratio of the dilation initiation stress to the rock strength follows a similar trend. This variation does not indicate a clear correlation between the dilation initiation stress of the mudstone and the loading rate. These results, combined with the data shown in Fig. 12, suggest that the dilation initiation strain has a certain power function relationship with the loading rate. In the literature, the dilation initiation stress obtained through stress-controlled methods does show a tendency to correlate with the loading rate. This variation may be related to the selected loading rates. The current loading rate is a strain-controlled loading rate, and therefore, the control method of the loading rate may influence the dilatancy characteristics of mudstone to some extent.

## Study of the acoustic emission characteristics of mudstone

During the rock loading and failure process, a significant amount of acoustic wave information is generated. Acoustic emission, a widely used nondestructive monitoring method, can accurately record changes in acoustic waves during the rock failure process^31–36^. In this study, acoustic emission was employed to analyse and study the ringing count, energy variations, crack morphology, and failure modes of mudstone under different loading rates.

### Acoustic emission ringing count characteristics

**Figure 14:**
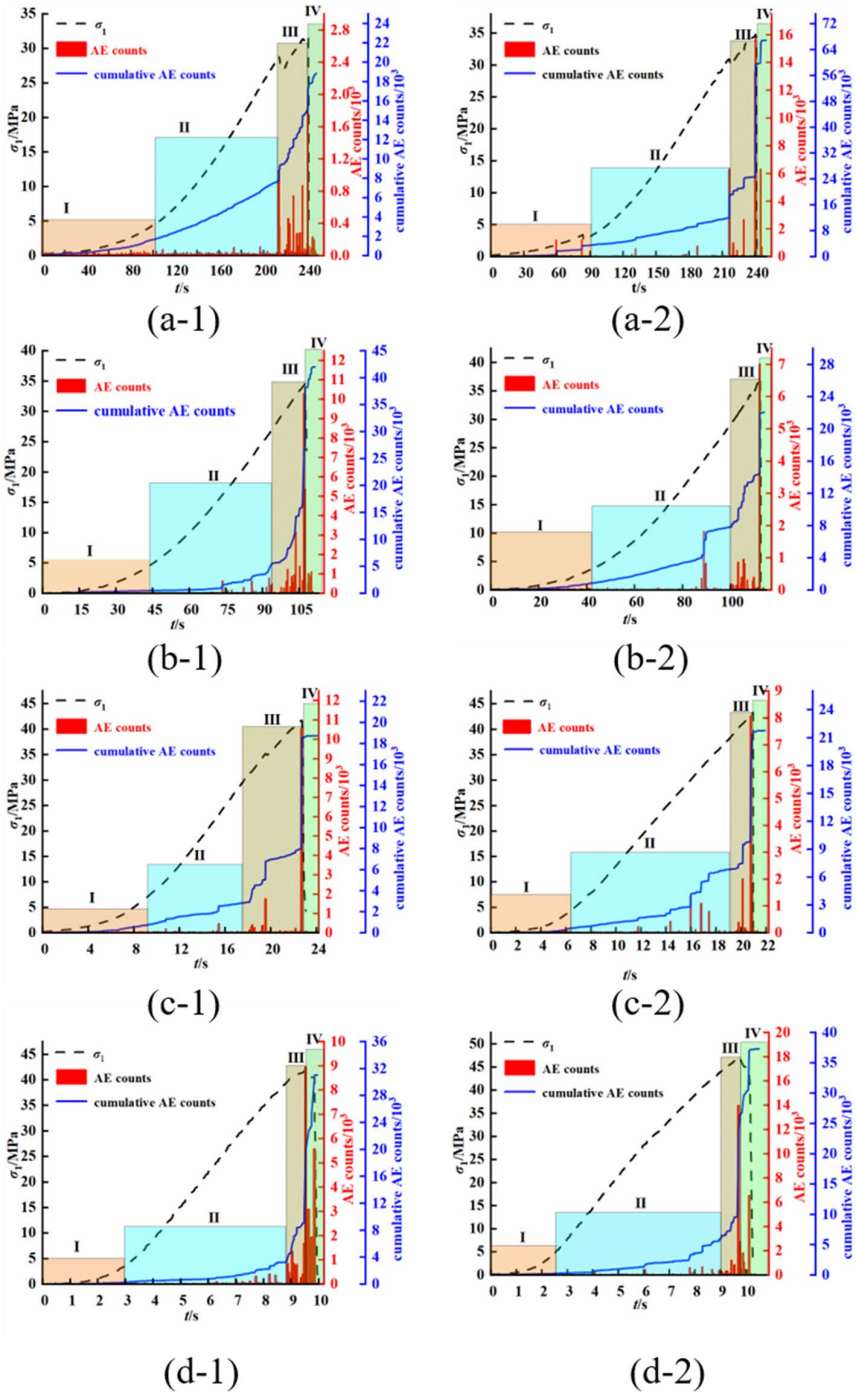
Acoustic emission ringing counts and cumulative ringing counts of mudstone under different loading rates (**a-1** and **a-2** show the results for a loading rate of 0.005 mm/s, **b-1** and **b-2** show the results for a loading rate of 0.01 mm/s, **c-1** and **c-2** show the results for a loading rate of 0.05 mm/s, and **d-1** and **d-2** show the results for a loading rate of 0.1 mm/s).

Figure 14 shows the acoustic emission characteristics of mudstone under different loading rates. According to the acoustic emission features, the process of mudstone failure can be divided into four stages: the initial compaction stage (I), the quasielastic stage (II), the crack growth stage (III), and the postpeak stage (IV). In the initial compaction stage, the internal fissures of the mudstone are in a compacted state, and the emitted acoustic emission signals are very weak. In the quasielastic stage, because the current stress is less than the starting stress of the crack, no new cracks are generated inside the mudstone, and the acoustic emission signals in this stage are relatively weak, with only a few events observed. Subsequently, the mudstone enters the crack growth stage, where the stress in the mudstone exceeds the stress required for internal crack propagation, leading to the emergence of numerous cracks. During this stage, many acoustic emission signals are generated, and as the stress increases, the acoustic emission signals significantly increase until the rock fails. After the rock fails, it enters the postpeak stage, during which there is a slight increase in the ringing count, indicating that some rock fractures form in this stage, but the formation of new cracks is limited.

Furthermore, with increasing loading rate, the acoustic emission signals of mudstone exhibit different behaviours in each stage. We define the ratio n as the proportion of the time when the mudstone acoustic emission ringing count is high to the total time until final failure. At a loading rate of 0.005 mm/s, the mudstone emits a small number of acoustic emission signals during the compaction stage. As loading progresses, the number of acoustic emission signals gradually increase. Before reaching the crack propagation stage, the acoustic emission signals remain at a relatively low level. Both sample groups experienced a significant increase in ringing count at approximately 210 s, with a current n value of approximately 0.8. When the loading rate is 0.1 mm/s, the time required for mudstone to enter the crack propagation stage is approximately 9 s, and the n value is approximately 0.9. With increasing loading rate, the n value of the mudstone gradually increases. This situation indicates that with increasing loading rate, the time from the appearance of many acoustic emission events to the failure of the mudstone decreases. This suggests that the failure mode of mudstone changes from a progressive process of microcrack propagation and penetration to a sudden and instantaneous failure process with increasing loading rate.

### Acoustic emission b-value and energy

The phenomenon of acoustic emission accompanying the rock failure process can be considered a small-scale earthquake. Thus, the b-value, introduced from seismology, has been incorporated into the study of rock failure acoustic emission. The characteristics of the change in b-value can reflect the scale of internal microcrack propagation in rocks^37–42^. Therefore, conducting research on acoustic emission b-values is of importance for understanding the intrinsic mechanisms of rock failure^43^.

The acoustic emission b-value is not widely used in the field of rock mechanics. Referring to the calculation method of b-values in seismology, this study adopts the Gutenberg–Richter (G–R) formula proposed by Gutenberg and Richter in 1941.

2 $${\text{logN}} = {\text{a}} - {\text{bM}}$$

where M is the magnitude, N is the number of events, and a and b are constants. b is used in the relative magnitude distribution function in earthquakes and is an important parameter in measuring seismic activity. In rock failure research, it represents the crack propagation scale function. Calculating the b-value of acoustic emission first requires determining the magnitude of the acoustic emission signals from rock samples. In this paper, the amplitude was used for conversion, and the conversion formula is as follows:

3 $${\text{M}} = \frac{{{\text{A}}_{{{\text{dB}}}} }}{{{20}}}$$

In the equation above, A_dB_ represents the maximum amplitude of the acoustic emission event measured in decibels, and A_dB_ = 20lgA_max_, where A_max_ is the maximum amplitude of the acoustic emission event measured in microvolts (μV).

**Figure 15:**
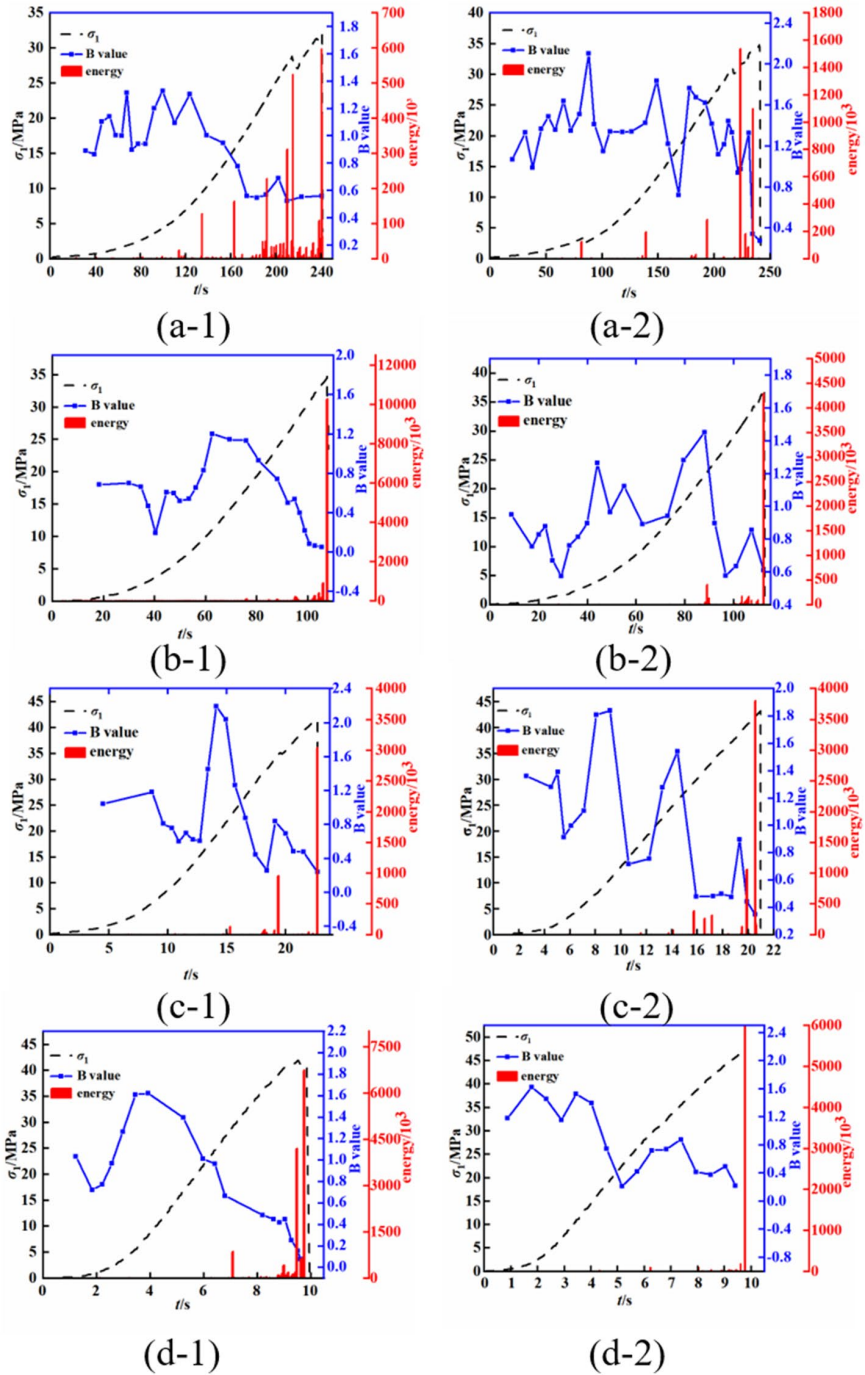
Acoustic emission b-values and energy of mudstone under different loading rates (**a-1** and **a-2** show the results for a loading rate of 0.005 mm/s, **b-1** and **b-2** show the results for a loading rate of 0.01 mm/s, **c-1** and **c-2** show the results for a loading rate of 0.05 mm/s, and **d-1** and **d-2** show the results for a loading rate of 0.1 mm/s).

After determining the calculation formula, the acoustic emission b-values of the mudstone were calculated using the least squares method, with a sliding window of 100 data points and a magnitude interval ΔM of 0.5. The acoustic emission b-values of the mudstone under different loading rates were obtained, as shown in Fig. 15.

The variation curves of the acoustic emission b-values are shown in Fig. 15. During the loading process, the acoustic emission b-values continuously change. In the initial compaction stage, the acoustic emission b-values fluctuate at a relatively high level, indicating that the predominant behaviour within the rock is the compaction of microcracks. As the loading progresses, the rock enters the elastic stage, characterized by fewer acoustic emission signals and small fluctuations in b-values. With ongoing loading, more energy signals from the rock are gradually recorded, and the b-values rapidly increase just before the onset of acoustic emission signals. When the energy signals are first recorded, the acoustic emission b-values decrease. This pattern primarily indicates the occurrence of internal crack propagation within the rock. As the loading continues, the rock gradually undergoes damage, with many acoustic emission energy signals appearing just before failure, leading to a gradual decrease in the acoustic emission b-values. In the early stages of loading, the acoustic emission b-values are higher, suggesting that the damage within the mudstone is dominated by the propagation of medium-sized to small cracks. In the later stages of loading, lower acoustic emission b-values are observed, indicating that the predominant mode of failure involves the propagation and connection of large cracks.

With increasing loading rate, the acoustic emission b-values of the mudstone exhibit varying degrees of change. At 0.005 mm/s, the acoustic emission b-values show small fluctuations in the early stages of loading. However, as the loading progresses, the number of acoustic emission events in the mudstone increases, leading to repetitive increases and decreases in the b-values. This process is associated with the expansion and connection of microcracks within the mudstone, indicating that shear failure is the primary mode of failure.

At a loading rate of 0.05 mm/s, the number of acoustic emission events in the mudstone decreases, and the b-values exhibit a sharp decline. This situation suggests that with an increase in the loading rate, the b-values of the mudstone change before failure. The mudstone damage transitions from the slow expansion and connection of microcracks observed at lower loading rates to a sudden and instantaneous failure. This is particularly evident at a loading rate of 0.1 mm/s, at which the mudstone exhibits fewer acoustic emission energy signals before failure, large energy signals appear in the final stages of failure, and the b-values show small-scale fluctuations. At this point, the failure of the mudstone is characterized by sudden and instantaneous rupture.

### Relationship between acoustic emission and crack morphology

Mudstone exhibits different internal crack patterns under various loading conditions, which are associated with distinct acoustic emission characteristics. Therefore, acoustic emission parameters can be used to characterize the extension of internal cracks in mudstone^44,45^. Numerous studies have confirmed that acoustic emission parameters (AF/RA combinations) can be employed to describe the types of cracks within rocks. The calculation formulas for AF and RA are as follows:

4 $${\text{AF}} = \frac{{{\text{AE}}\;{\text{counts}}}}{{{\text{duration}}}}$$

5 $${\text{RA}} = \frac{{{\text{rise}}\;{\text{time}}}}{{{\text{amplitude}}}}$$

In previous studies, a threshold for AF/RA has been defined to determine the types of cracks in rocks. When AF/RA is greater than a certain value, it is identified as a shear crack, and when AF/RA is less than a certain value, it is considered a tensile crack. Cracks falling between these thresholds are classified as mixed-mode cracks^46,47^. Analogous to the ratio of high and low frequencies under compressive conditions, tensile and shear crack AF (kHz):RA (ms/V) reference ratios have been established for brittle rock, typically ranging from 1:100 to 1:500. Kong^48^ used AF (kHz) = 200RA (ms/V) to analyse crack types in volcanic rocks, while Hu^49^ employed AF (kHz) = 70 RA (ms/V) as a crack classification reference line for analysing the ratio of tensile to shear cracks under conditions of rock bursts. Du^50^ studied the fracture process of different rock types and proposed that the crack classification reference line for sandstone is AF (kHz) = 3RA (105 μs/V) + 75. Meng^51^ studied crack types based on Brazilian splitting tests and normalized the AF/RA values, They suggested that the AF/RA threshold for tensile cracks in sandstone is 1.

The classification criteria for crack types in the rock fracturing process are still uncertain. Even for rocks of the same type with minor differences in lithology, the proportions of these microcrack classification reference lines are different. This variability may be related to the intrinsic properties of the rocks and the type of acoustic emission monitoring equipment. Therefore, the ratios of AF (kHz) to RA (ms/V) obtained in various studies for microcrack types range between 10.1:1 and 104:1.

**Figure 16:**
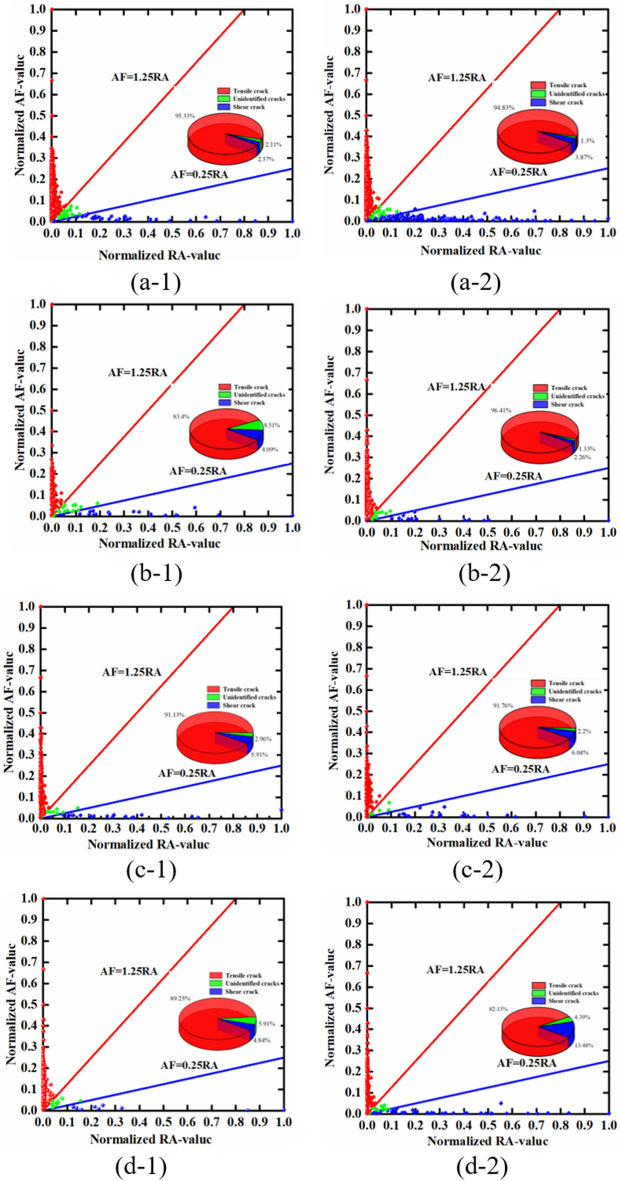
Normalized AF/RA values of mudstone acoustic emission under different loading rates (**a-1** and **a-2** show the results for a loading rate of 0.005 mm/s; **b-1** and **b-2** show the results for a loading rate of 0.01 mm/s; **c-1** and **c-2** show the results for a loading rate of 0.05 mm/s; and **d-1** and **d-2** show the results for a loading rate of 0.1 mm/s).

In this study, we normalized AF/RA and, following the classification standards in the literature, considered ratios between AF (kHz) and RA (ms/V) in the range of 0.25–1.25 as indicative of mixed-mode cracks, ratios greater than 1.25 as indicative of shear cracks, and ratios less than 0.25 as indicative of tensile cracks. The crack propagation characteristics of mudstone under different loading rates were analysed, and the specific experimental results are shown in Fig. 16.

Through normalization, some instrumental errors in the data can be effectively avoided. Figure 16 shows the types and proportions of fractures in the mudstone failure process under different loading rates. It is evident that the predominant failure mode of mudstone under different loading rates is tensile failure, characterized by the expansion and connection of tensile cracks. The accompanying shear cracks and mixed-mode fractures contribute to a lesser extent. As the loading rate increases, the proportion of tensile cracks in the mudstone gradually decreases, while the proportion of shear cracks increases. At a loading rate of 0.005 mm/s, tensile cracks account for an average of 95.08% of the mudstone failure, whereas shear cracks account for an average of 3.22%, and mixed-mode fractures account for an average of 1.71%. When the loading rate is increased by 2, 10, and 20 times, the average proportion of tensile cracks decreases by 5.18, 3.64, and 9.39%, respectively. Moreover, the average proportion of shear cracks increases by 2.13, 2.76, and 6.12%, respectively. This change indicates that with increasing loading rate, the number of tensile cracks gradually decreases, and the proportions of shear cracks and mixed-mode fractures increase. The failure mode of the mudstone gradually transitions from tensile failure to shear failure with increasing loading rate. However, the predominant form of mudstone failure still involves the expansion and connection of tensile cracks, resulting in tensile failure.

### Rock failure modes

**Figure 17:**
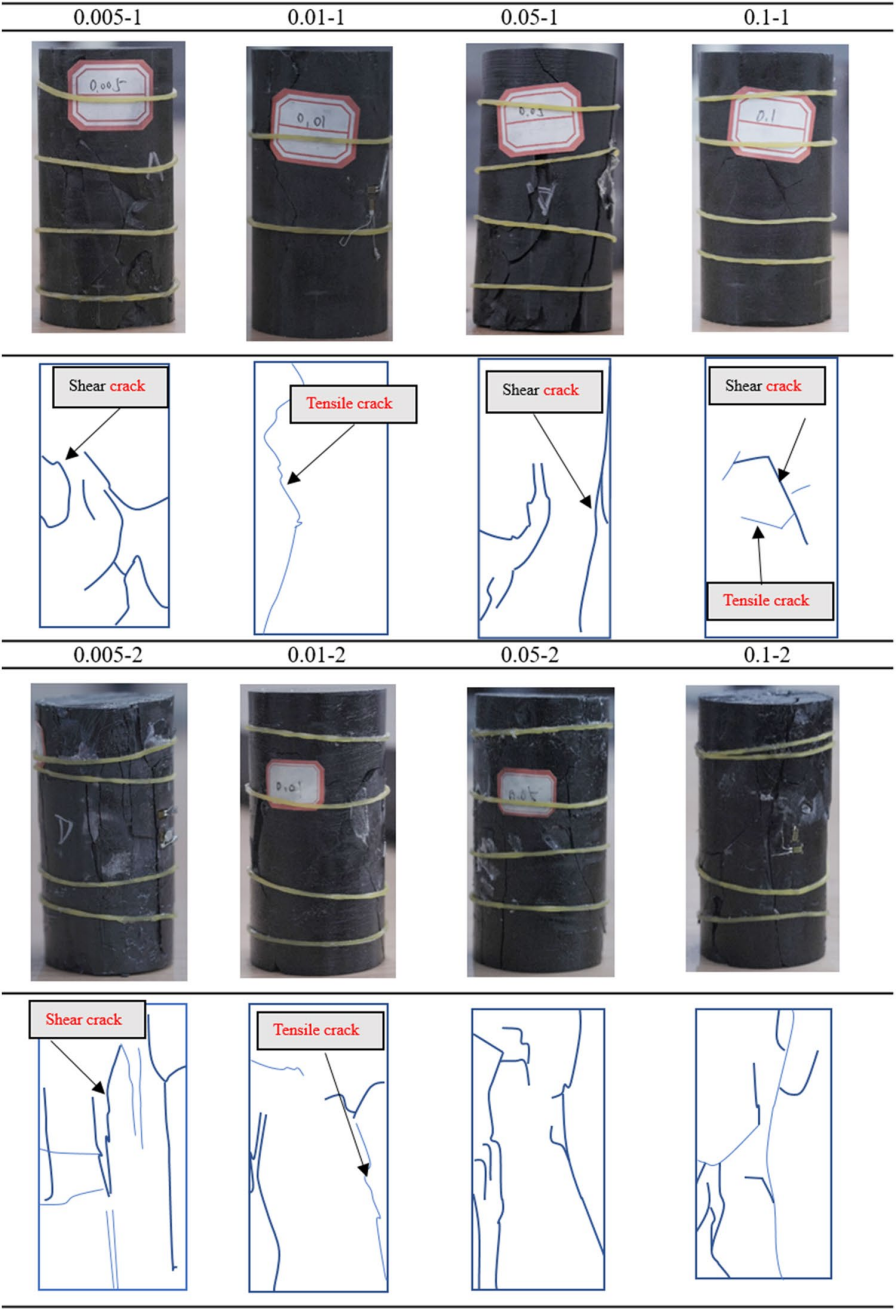
Failure modes of mudstone under different loading rates.

Figure 17 shows the crack propagation characteristics of mudstone under different loading rates. These results, combined with the acoustic emission results, clearly show that the failure mode of mudstone is characterized by primarily shear failure, accompanied by the extension of tensile and mixed cracks. The failure characteristics of mudstone are quite complex. When the loading rate is 0.005 mm/s, as for specimen 0.005–1, due to the relatively low loading rate, stress is uniformly transmitted inside the mudstone, and the failure is mainly dominated by the extension and penetration of shear cracks. In specimen 0.005–2, numerous vertical tensile cracks appear, indicating a predominant tensile failure mode with a significant amount of shear failure. When the loading rate is 0.01 mm/s, there are fewer cracks in both groups of specimens, and the main failure mode is shear failure. With increasing loading rate, the mudstone failure mode consistently involves the dominance of shear cracks, accompanied by the extension of tensile cracks. However, the failure mode of mudstone does not show a consistent trend with increasing loading rate, indicating that the failure mode of mudstone is relatively insensitive to the loading rate. As the loading rate increases, the number of cracks in the mudstone gradually decreases, indicating a transition from the gradual expansion of microcracks to sudden failure with the development of large cracks.

The acoustic emission characteristics of mudstone under different loading rates indicate that the failure mode of mudstone transitions from the gradual process of microcrack expansion and penetration to sudden and instantaneous failure as the loading rate increases. The transformation of this failure mode is related to the mineral composition and pore structure of the rock. Due to the presence of kaolinite in the rock, the bonding strength of the mudstone is lower that that of other sedimentary rocks, and there are more pore structures in the mudstone, resulting in strong randomness in the crack propagation of the mudstone under different loading rates. In this process, the occurrence of acoustic emission signals becomes more concentrated, approaching the moment of failure. This suggests that in engineering practice, under conditions of higher loading rates, the precursor information before the instability and failure of mudstone will not be as obvious and clear as that under lower loading rates. This makes the corresponding prediction of failure more challenging.

## Evolution of damage characteristics in shale under different loading rates

From a microscopic perspective, the failure of rock under uniaxial compression conditions involves the continuous propagation of internal microcracks leading to failure, ultimately resulting in overall failure. Simultaneously, energy storage and release occur throughout the loading process, conforming to the characteristics of material damage. Numerous experts have found that acoustic emission can be used assess the stress state and extent of damage in rocks. Assuming a uniform force on the rock sample, the damage degree of the mudstone can be expressed as follows:

6 $${\text{D}} = \frac{{{\text{S}}_{{\text{d}}} }}{{{\text{S}}_{{0}} }} = \frac{{{\text{N}}_{{\text{m}}} }}{{\text{N}}}$$

**Figure 18:**
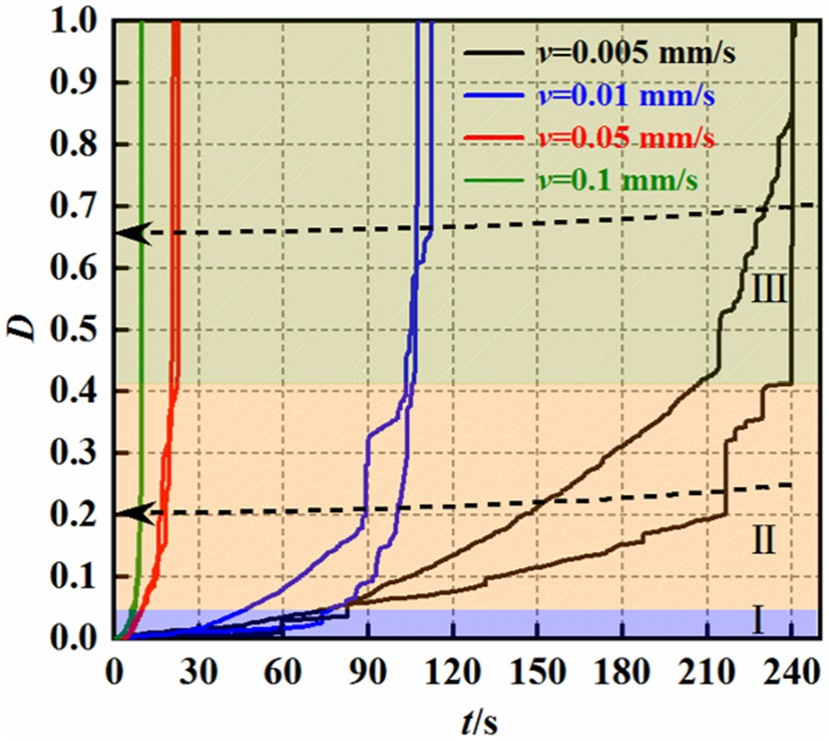
Evolution curves of the damage variables over time under the different loading rates.

The damage variable D can be defined as the ratio of the damaged area s_d_ to the initial undamaged area s_0_. In acoustic emission monitoring, the cumulative acoustic emission ringing count N_m_ can be used in relation to the total cumulative ringing count N to represent the damage. In this study, the damage extent of shale under different loading rates was investigated, and the results are presented in Fig. 18.

As shown in Fig. 18, the mudstone damage under the different loading rates can be roughly divided into three stages. In the stress‒strain curve, the first stage corresponds to the initial compaction process of mudstone, where internal cracks are gradually compacted, resulting in relatively minor damage. As loading progresses, the mudstone enters the elastic stage, during which the microcracks begin to expand, leading to an increase in the damage variables. When the stress‒strain curve enters the plastic stage, the number of microcracks inside the mudstone gradually increases, and crack expansion becomes pervasive. In this stage, the damage degree increases rapidly, exhibiting an exponential growth pattern.

With increasing loading rate, the three stages of mudstone damage gradually become more difficult to delineate. Under high loading rates, the first and second stages of mudstone deformation are extremely short, indicating rapid damage development. This characteristic suggests that under high loading rates, the failure of mudstone is instantaneous and abrupt. The reason for this trend may be that the increased loading rate results in insufficient internal stress transfer within the mudstone, causing microcracks to react too quickly, leading to an immediate failure state. This outcome poses significant challenges for the prediction of mudstone failure in practical engineering.

## Conclusion

Through in situ monitoring of acoustic emissions during uniaxial compression tests on mudstone at different loading rates, the impact of loading rate on the mechanical properties, failure mode, and damage behaviour of mudstone was revealed. The main conclusions are as follows:

1. With increasing loading rate, the elastic modulus and strength of mudstone increase similarly, but the magnitudes of increase change with increasing loading rate. The loading rate can influence only the peak strength and elastic modulus of mudstone to a certain extent, as the influence decreases with increasing loading rate.
2. According to the mudstone dilation characteristics, with increasing loading rate, the maximum volumetric strain of the mudstone gradually decreases, and the magnitude of decrease gradually decreases, approaching a constant value. This suggests that the influence of the loading rate on the volumetric strain is limited and tends to decrease gradually with increasing loading rate.
3. With increasing loading rate, the predominant failure mode of mudstone remains the extension and penetration of shear cracks, accompanied by the extension of tensile cracks. However, there is no consistent change in the failure mode of mudstone with increasing loading rate, indicating that the failure mode of mudstone is relatively unaffected by the loading rate. As the loading rate increases, the number of cracks during the failure process of mudstone gradually decreases, indicating a transition from the extension of microcracks to sudden failure with the development of large cracks. The division of the mudstone damage evolution into three stages becomes increasingly difficult to identify with increasing loading rate. Under high loading rates, the durations of the first and second stages are extremely short, indicating the rapid development of damage and failure in mudstone. This characteristic suggests that under high loading rates, the failure of mudstone is instantaneous and abrupt.

## Data Availability

The data that support the findings of this study are available from the corresponding author upon reasonable request.
